# A rare case of malignant hypertension with splenic rupture and thrombotic microangiopathy: A case report

**DOI:** 10.1097/MD.0000000000020581

**Published:** 2020-07-10

**Authors:** JiaXiang Ding, Zhen Qu, Feng Yu

**Affiliations:** a Renal Division, Department of Medicine, Peking University International Hospital; b Renal Division, Department of Medicine, Peking University First Hospital; Institute of Nephrology, Peking University; Key laboratory of Renal Disease, Ministry of Health of China; Key laboratory of CKD prevention and treatment, Ministry of Education of China; Beijing, P.R. China.

**Keywords:** malignant hypertension, spontaneous splenic rupture, thrombotic microangiopathy

## Abstract

**Introduction:**

Thrombotic microangiopathy (TMA) is characterized by endothelial injury followed by formation of multiple microthrombi in the target organs due to various causes, including malignant hypertension (MHT). Here, we reported a rare case of MHT with splenic TMA changes.

**Case concerns:**

A 27-year-old Chinese Han male with a history of hypertension and proteinuria, admitted to our hospital because of renal failure with MHT and thrombocytopenia.

**Diagnosis:**

This case diagnosed with TMA based on the patient's MHT and thrombocytopenia. The patient final diagnosis was confirmed by the spleen pathological findings, other differential diagnoses were ruled out.

**Interventions:**

The patient was treated with hemodialysis and intravenous antihypertensive agents, and his condition gradually improved. However, he suddenly complained of abdominal pain and went into hemorrhagic shock, which was due to spontaneous spleen rupture on the third day of hospitalization. The pathological evidence after splenectomy showed splenic TMA.

**Outcomes:**

Hemodialysis was continued and the blood pressure was under control until his discharge from our hospital.

**Conclusion:**

Spontaneous splenic rupture could be a rare and critical complication associated with MHT-induced TMA, and it requires careful clinical attention.

## 1 Introduction

Malignant hypertension (MHT) is a clinical syndrome characterized by severe hypertension and organ damage, such as progressive renal failure, heart failure and encephalopathy.^[1]^ The pathological changes associated with MHT include endothelial injury, arteriolar intimal hyperplasia, glomerular ischemia, and sclerosis in the chronic phase, and so on. MHT could also cause thrombotic microangiopathy (TMA) changes.^[2,3]^ Herein, we reported a rare case of MHT inducing splenic TMA, proven by the pathological study of the spleen conducted after the splenectomy of the patient.

## 2 Case presentation

A 27-year-old Chinese Han male was admitted to the emergency room of our hospital with a chief complaint of nausea for 1 week, anuria for 3 days, and shortness of breath for 1 day.

He had a history of hypertension for 3 years and his highest blood pressure (BP) raised up to 180/120mm Hg. At the same time, proteinuria was noted, and his renal function had decreased with serum creatinine value at 1.5 mg/dL and estimated glomerular filtration rate (eGFR) at 63 mL/min·1.73m^2^. Ultrasound results revealed that there were no abnormalities regarding the kidney size, renal arteries and adrenal gland. The patient took nifedipine and valsartan to control the BP at 120/70mm Hg for 2 years without any renal evaluation since then. Two months ago, he discontinued his anti-hypertension drugs because of a business travel. He had no history of anemia, thrombocytopenia, or other hematological diseases.

During admission, his BP was 214/132 mm Hg, pulse 86 beats/min, respiratory rate 20 breaths/min, and body temperature 36.5°C. Chest auscultation revealed scattered crackles and bilateral edema was found in lower extremities.

The urine examination showed that urine protein was 3+, red blood cell count 3140 cells/μL, and white blood cell count 8 cells/μL. The whole blood cell analysis revealed white blood cell 8.42  × 10^9^/L (3.5–9.5 × 10^9^/L), neutrophil 78.3% (40–75%), hemoglobin 88 g/L (130–175 g/L), and platelet count 43 × 10^9^/L (125–350 × 10^9^/L). The serum creatinine value was 26.6 mg/dL (0.67–1.18 mg/dL), blood urea nitrogen was 54.79 mmol/L (1.7–8.3 mmol/L), and potassium level was 5.86 mmol/L (3.5–5.3 mmol/L). The lactate dehydrogenase level was raised at 2158 U/L (120–250 U/L) and Coombs’ test was negative. There was no evidence of increased schistocytes in the peripheral blood smear. Hepatic function, serum bilirubin, prothrombin time, activated partial thromboplastin time, and serum fibrinogen and fibrinogen degradation product levels were all within normal ranges. The level of brain natriuretic peptide was 349 pg/mL (<100 pg/mL). Results for tests of antinuclear antibodies, antineutrophil cytoplasmic antibodies, C3 and C4 complemnt levels were normal. His ADAMTS13 (A Disintegrin And Metalloprotease with a ThromboSpondin type 1 motif, member 13) activity was 74% (40–99%). The plasma factor H concentration was 418 μg/mL (247–1010 μg/mL) and auto-antibody against factor H tested negative. Fundus examination revealed retinal hemorrhage, soft exudates and copper wire arterioles.

The patient was initially diagnosed with MHT and acute kidney injury along with chronic kidney disease. Because of the bilaterally decreased kidney size by observed on ultrasound examination, the renal biopsy was not formed. Hemodialysis was initiated and intravenous urapidil hydrochloride was infused. The BP decreased gradually to 160/110 mm Hg and the symptoms of nausea, vomiting, shortness of breath improved within 2 days.

**Figure 1:**
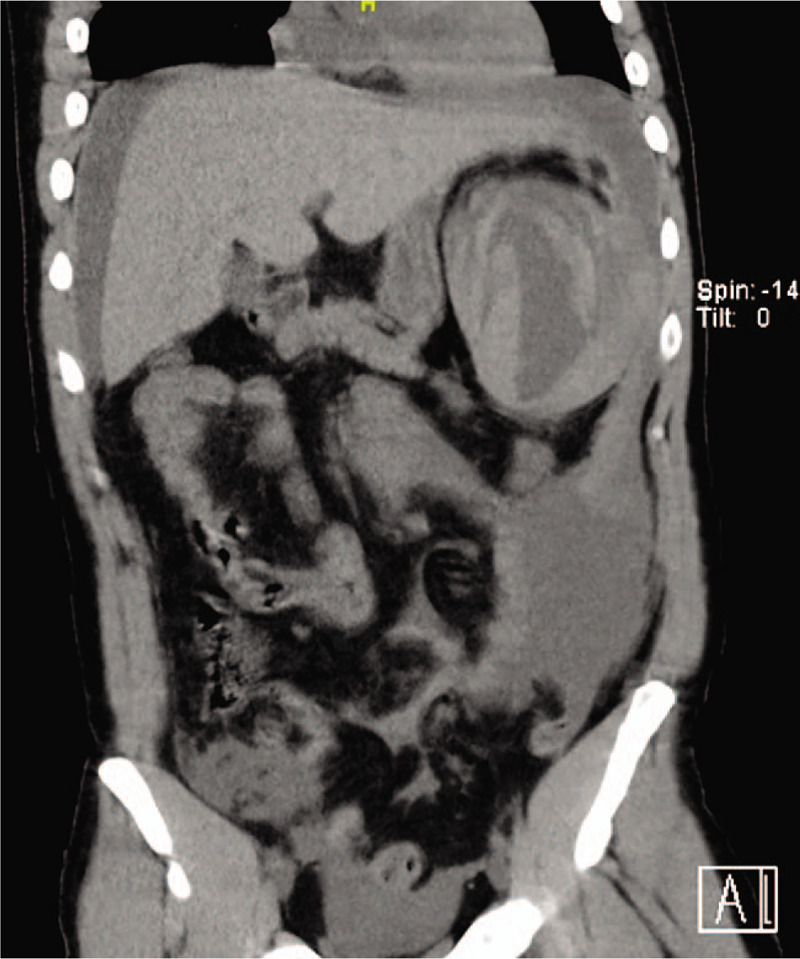
Computed tomography scan showed shows that the spleen was is enlarged and ruptured. The arrow denotes that there were are multi-focal hemorrhages in the capsule and parenchyma.

On the third day after admission, the patient suddenly complained of pain in his left upper quadrant region after he went to the toilet. His stool color was normal, BP dropped to 134/80 mm Hg, heart rate increased to 101 beats/min, and breathing rate was 20 times/min. The physical examination revealed paleness of skin, mild tenderness in the upper abdominal without rebound tenderness, and weak bowel sounds. Laboratory test showed that the hemoglobin had decreased from 81 g/L to 35 g/L within 4 hours. Hence, acute hemorrhage of unknown causes was suspected. The abdominal ultrasonography showed mixed echo in the lower splenic range and liquid in the pelvic cavity. Abdominal cavity puncture was performed immediately revealing blood in the pelvic cavity. The computed tomography (CT) scan proved splenic rupture that caused the hemorrhage under the capsule of the spleen (Fig. 1).

**Figure 2:**
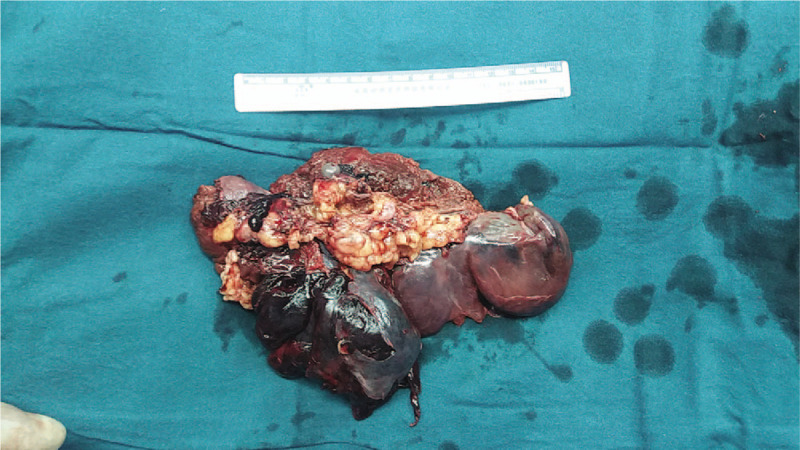
The gross appearance of the ruptured spleen after splenectomy. The spleen size is 15 × 9 × 5 cm and the outer membrane of the spleen was being torn extensively with massive hemorrhage.

Exploratory laparotomy was performed that revealed that the outer membrane of the spleen was torn extensively with massive hemorrhage (Fig. 2). The splenectomy was then performed because the splenic rupture was not repairable, and the patient was transferred to the intensive care unit ward after surgery.

**Figure 3:**
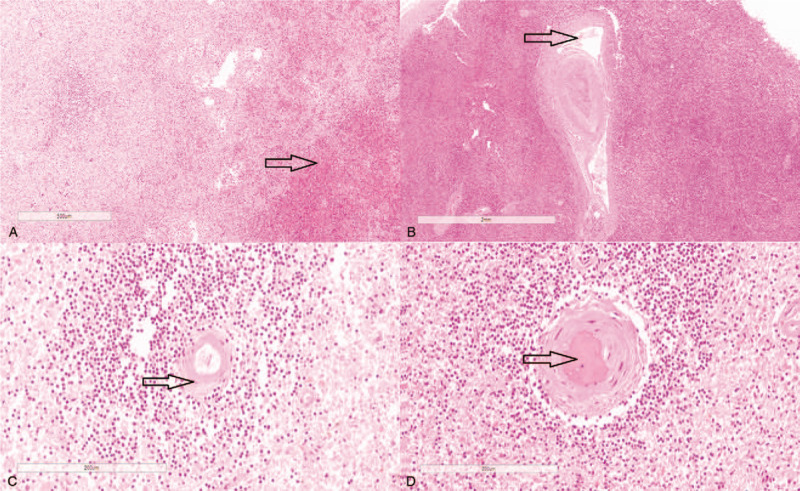
Histological presentations of the the spleen pathology presentations. Note: (A) Massive hemorrhage and necrosis is observed in the parenchyma with several lymphocytes and neutrophils infiltrations in the splenic membrane (H&E X50). (B) There were is yaline degeneration in the small artery wall with middle wall ruptured and intercalation (H&E X20). (C) Focal infiltrations of lymphocytes and neutrophils is observed in some vessel walls with intimal thickening (H&E X200). (D) Thrombosis and partial small vessel occlusion is observed (H&E X200). (H&E: Hematoxylin and eosin stain).

The splenic pathology showed multi-focal hemorrhages in the capsule and the parenchyma had multiple lymphocytic and neutrophilic infiltrations (Fig. 3A). In some vessel walls, there were hyaline degeneration and focal fibrous necrosis. Thrombosis was noted within some small arteriolar lumens. There were lymphocytes and neutrophilic infiltrations around the vessels and some arteries showed intimal thickening, middle wall rupture and intercalation (Fig. 3B, C, D). Splenic arteriolar hyalinization confirmed the suspicious of early response of the vascular wall to elevated mechanical pressure. In combination with the clinical presentations, it was believed that the spontaneous splenic rupture could be related to MHT, which caused the TMA like lesions.

Thirteen days after the operation, the abdominal CT was re-evaluated and no bleeding was found around the splenic area. Two months after the operation, the patient was discharged from our hospital with BP under control, but hemodialysis was continued.

## 3 Discussion

MHT is characterized by severe hypertension and multi-organ ischemic complications including TMA.^[3,4]^ The diagnostic criterion of MHT includes diastolic BP ≥120 mm Hg, along with ocular hemorrhages and exudates with papilledema (grade IV Kimmelstiel-Wilson retinopathy),^[5,6]^ and TMA should be diagnosed by decreased platelets (typically  < 10–30 × 10^9^/L), microangiopathic hemolytic anemia (typically 80–100 g/L), with schistocytes (typically > 1%) in the peripheral blood smear, elevated lactate dehydrogenase, and multiple organs dysfunction, such as kidneys.^[7,8]^ The spleen has been rarely reported to be involved in MHT-induced TMA with confirmed evidence of splenic histology, as seen in the case reported here. Our patient fulfilled both the MHT and TMA criteria, especially based on the splenic histologic evidence.

MHT is a clinical syndrome caused by a variety of causes.^[2]^ Nearly 20% to 40% of MHT cases are caused by primary hypertension,^[9]^ and secondary MHT refers to renal parenchymal diseases, renal vascular diseases, endocrine diseases, drug induced hypertension, and so on. Renal parenchymal disease is the main reason for MHT, and IgA nephropathy was most common in some observational studies.^[10]^ With the exclusion of the adrenal gland disease and renal artery stenosis by CT and Doppler flow imaging examinations, our young patient could have the chronic glomerulonephritis based on his past history of proteinuria although the renal pathology was not performed.

The patient also presented with TMA which could be associated with MHT. Although TMA could be complication of MHT,^[11]^ it could also “act” as 1 of the clinical features of TMA, like complement associated atypical hemolytic uremic syndrome.^[12]^ Recently, Sameg et al evaluated the role of complement in 9 consecutive patients with biopsy-proven renal TMA attributed to severe hypertension, and found mutations *C3* in 3, *CFI* in 1, *CD46* in 1, and/or *CFH* in 2.^[13]^ Thus, further explorations on complement, including their genetic mutations and circulation activation status is needed for such cases in the future.

More importantly, spontaneous splenic rupture occurred in this patient after his admission despite well controlled BP. Spontaneous splenic rupture is rare, and accounts for 3% to 4% of the total splenic ruptures.^[14]^ It has varied pathogenesis and etiologies, such as infectious diseases, splenic occupying diseases, hematological diseases, drug-induced events, and splenic artery aneurysm.^[14–16]^ Our case was the first with pathologically-proven splenic TMA induced by MHT and based on the direct pathological characteristics of the spleen, such as hyaline degeneration in the small artery wall, focal fibrous necrosis, thrombosis, and partial small vessel occlusion. In theory, MHT could damage the micro-endothelial cells of viscera such as spleen by elevated shear stress, inflammation, over-activation of complement, aberrant coagulation “storm” and so on, besides the more often targeted organs like kidney, brain and heart.^[17,18]^ From the splenic pathology, we might infer inferred that MHT and TMA were the potential pathological basis for splenic rupture.

In conclusion, spontaneous splenic rupture could be a rare and critical complication associated with MHT-induced TMA and it requires careful clinical attention.

## Acknowledgments

Authors thank everyone that contributed to this study.

## Author contributions

**Supervision:** Feng Yu.

**Writing – original draft:** JiaXiang Ding.

**Writing – review & editing:** Zhen Qu.
